# Total synthesis of (±)-simonsol C using dearomatization as key reaction under acidic conditions

**DOI:** 10.3762/bjoc.21.47

**Published:** 2025-03-17

**Authors:** Xiao-Yang Bi, Xiao-Shuai Yang, Shan-Shan Chen, Jia-Jun Sui, Zhao-Nan Cai, Yong-Ming Chuan, Hong-Bo Qin

**Affiliations:** 1 School of Chemistry and Environment, Yunnan Minzu University, Kunming 650000, China

**Keywords:** acidic dearomazation, benzofuran, (±)-simonsol C, total synthesis

## Abstract

The total synthesis of (±)-simonsol C was accomplished using a dearomatization under acidic conditions as key step to construct an aryl-containing quaternary center. The 6/5/6 benzofuran unit was formed through reductive elimination with Zn/AcOH and a spontaneous oxy-Michael addition. This synthesis consists of 8 steps and achieves an overall yield of 13%, making it the shortest known route.

## Introduction

**Figure 1:**
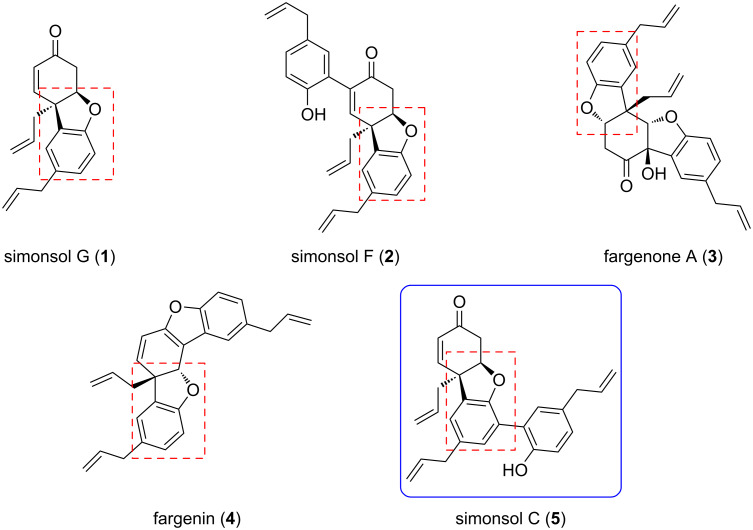
Representative sesquineolignan compounds.

Star anise, derived from *Illicium* species cultivated in southeastern China [1] possesses significant economic, culinary, and medicinal value [2]. Particularly noteworthy are its medicinal properties, including insecticidal, antibacterial, anti-inflammatory, analgesic, and neurotrophic activities [3]. In 2013, Wang’s group isolated (±)-simonsol C from star anise, which features a unique 6/5/6 tricyclic benzofuran structure [4]. They found that it exhibits biological activity that promotes neuronal synapse growth and inhibits acetylcholinesterase.

(±)-Simonsol C (Figure 1) has received considerable attention due to the presence of an aryl- and allyl-containing quaternary carbon center, which is common in natural products such as galanthamine and morphine. To construct the quaternary carbon in simonsol C, two reports have utilized alkaline dearomatization strategies and another report used an intramolecular Heck reaction as the key reaction [5–7]. However, there have been no reports or studies utilizing acidic dearomatization, which is also effective, to synthesize an arylated quaternary carbon center.

**Scheme 1:**
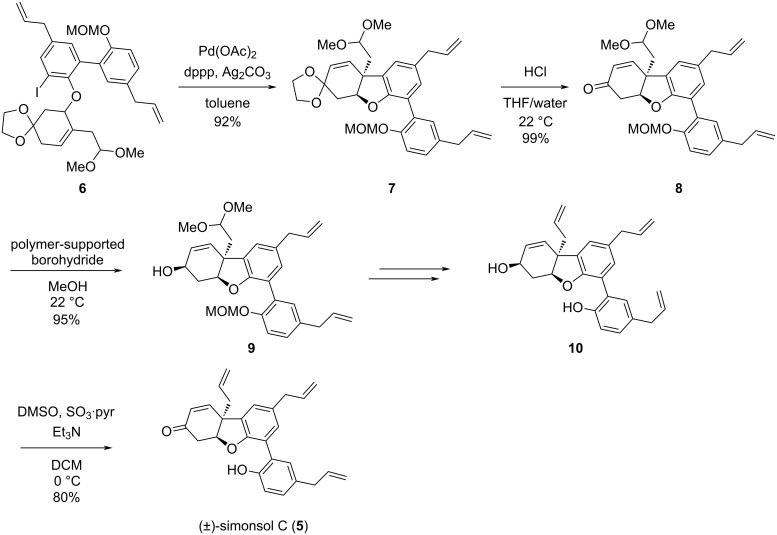
The first total synthesis of (±)-simonsol C by Banwell’s group.

In the first report on the total synthesis of simonsol C (Scheme 1), in 2016 Banwell’s group employed an intramolecular Heck reaction as key step to furnish the aryl-containing quaternary center and simultaneously construct the benzofuran skeleton [7]. This synthesis involved a total of 12 steps and achieved 12% overall yield.

**Scheme 2:**
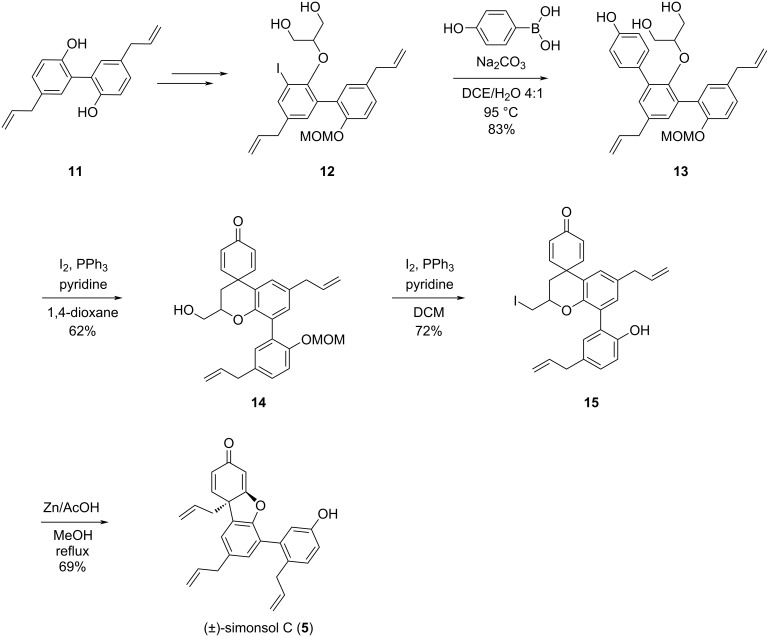
The second total synthesis of (±)-simonsol C developed by the Qin group.

In May 2024, the Qin group reported the second total synthesis of (±)-simonsol C (Scheme 2) [5]. An effective strategy to form the 6/5/6 benzofuran scaffold was developed which specifically involved a basic dearomatization and reductive elimination with Zn/AcOH to construct the aryl and allyl-containing quaternary center, and a simultaneous phenol-initiated oxy-Michael addition to afford the benzofuran unit. This synthesis took 9 steps and achieved an overall yield of 13%. Also in 2024, the Denton group reported another efficient way to access the 6/5/6 benzofuran scaffold of simonsol C, utilizing an alkaline dearomatization as the key reaction, followed by a functional-group-selective Wittig reaction and concurrent oxy-Michael addition [6]. A bromophenol acetal was used in the intramolecular alkylative dearomatization, which was first reported by Magnus et al. [8] and has been used in syntheses of natural products containing aryl quaternary carbon centers [9–10].

Unlike the intramolecular alkylation strategy of a phenol derivative, which can only be applied in basic dearomatization reactions, our approach using an α-iodophenol ether as precursor of the dearomatization offers considerable versatility. Not only can it be employed under basic dearomatization conditions, but it is also effective under Lewis acid conditions. Combined with a reductive elimination using Zn/AcOH, the benzofuran skeleton can be easily synthesized. This dual applicability of the new approach will be demonstrated next in the synthesis of simonsol C.

## Results and Discussion

**Figure 2:**
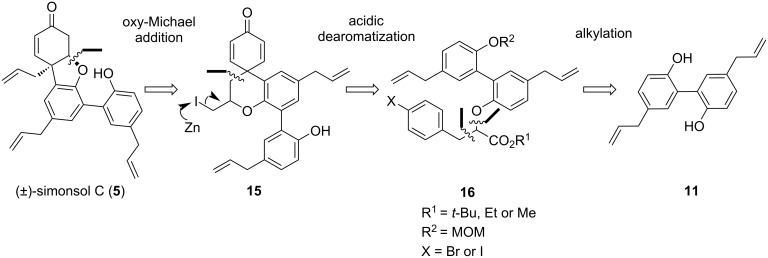
Retrosynthetic analysis of (±)-simonsol C.

Based on extensive literature investigations, the retrosynthetic analysis strategy for our synthesis of (±)-simonsol C is as follows (Figure 2): The 6/5/6 benzofuran skeleton of (±)-simonsol C can be accessed via an oxy-Michael addition from dienone **15**. The 6/6/6 tricyclic structure in **15** can be constructed through dearomatization of compound **16**, which in turn can be readily synthesized through consecutive alkylation steps starting from magnolol (**11**). Additionally, using magnolol as the starting material brings two allyl groups into the product, thus avoiding the challenges associated with allyl formation reactions.

**Scheme 3:**
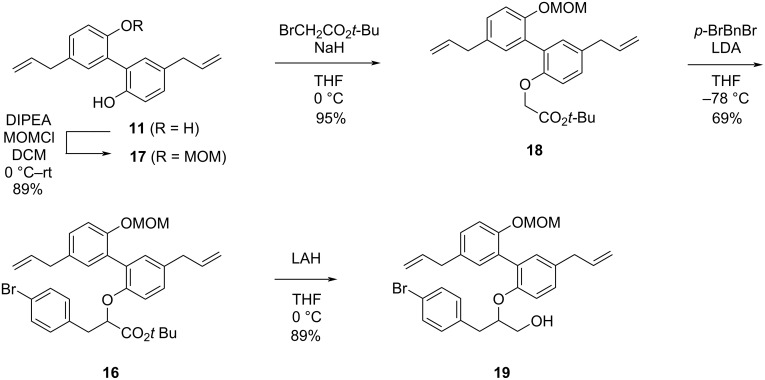
Rapid access of the basic skeleton of (±)- simonsol C.

The chosen synthetic route towards (±)-simonsol C is shown in Scheme 3. Starting with magnolol (**11**), one of the phenol groups was selectively protected by controlling the equivalents of MOMCl and DIPEA, affording compound **17** with an 89% yield [11].

For the following alkylation step with *tert-*butyl bromoacetate, three bases were tested: potassium carbonate, cesium carbonate, and sodium hydride. Considering the targeted alkylation of a phenolic hydroxy group and the p*K*_a_ requirements of this reaction, weaker bases like potassium carbonate and cesium carbonate should theoretically suffice. However, the reaction outcomes with these two bases did not meet the desired expectations, as some starting material remained after 5 hours of reaction. Extending the reaction time did not lead to full consumption of the starting material. Subsequently, when the base was changed to the stronger base sodium hydride [12], the reaction proceeded much better. Within 2 hours, the starting material was completely converted, yielding compound **18** with 95% isolated yield.

Proton abstraction of the hydrogen in the α-position to the carbonyl group in **18** was achieved by using LDA, followed by the addition of 4-bromobenzyl bromide for alkylation, giving compound **16** with 69% isolated yield. Compound **16** was then reduced to alcohol **19** with 2 equiv LAH at 0 °C. The reaction was completed within 10 minutes and the desired alcohol **19** was isolated in 89% yield.

**Scheme 4:**
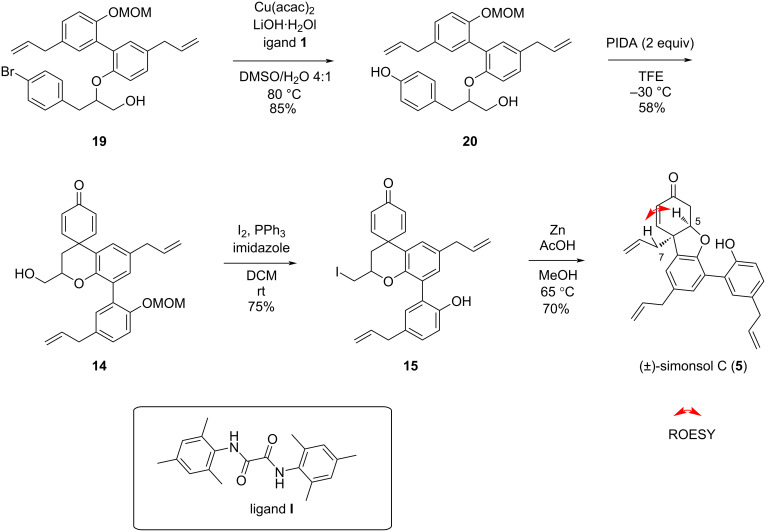
Synthetic details to (±)-simonsol C.

The copper-catalyzed replacement of the bromine substituent in **19** with a hydroxy group was achieved in the presence of a catalytic amount of oxalamide ligand **I** [13]. This transformation is critical for enabling further functionalization and the reaction conditions were optimized to achieve product **20** with 85% yield, minimizing potential side reactions. The subsequent dearomatization step is crucial for the construction of the cyclohexadienone unit. Oxidation of compound **20** with PIDA in trifluoroethanol, the original phenol was converted into a quinone moiety, successfully forming the aryl-containing quaternary center. However, in this step, the reaction was too rapid to control. After optimizing the reaction time and temperature, the reaction was carried out at −30 °C for 15 minutes and product **14** was isolated in a yield as high as 58% [14]. Iodination of compound **14** was performed next and the desired iodide was isolated and, to our delight, the cleavage of the MOM group occurred concomitantly, affording compound **15** in 75% yield. This reaction is likely triggered by the in situ-generated acid. As in our previously reported synthesis, a Zn/AcOH reductive elimination was utilized to liberate the allyl group and to simultaneously construct the 6/5/6 tricyclic skeleton via an oxy-Michael addition affording (±)-simonsol C in 70% yield (Scheme 4). The spectral data were in agreement with the reported ones [4,15–16] and the *cis* relation between the protons at C5 and C7 in simonsol C was confirmed by ^1^H-^1^H ROESY spectroscopy.

## Conclusion

The total synthesis of (±)-simonsol C was accomplished using a dearomatization reaction under acidic conditions as key step to construct the aryl-containing quaternary center. The 6/5/6 benzofuran unit was formed through reductive elimination with Zn/AcOH and spontaneous oxy-Michael addition. This route largely enhances the synthetic efficiency and shortens the number of synthetic steps. The whole synthesis route involves 8 steps and affords the final product in a total yield of 13%, which could be the shortest synthesis route to date.

The structural motif of an all-carbon quaternary center containing an aryl group is common in many natural products, such as galanthamine and morphine. Our current strategy provides an alternative approach for the synthesis of aryl-containing quaternary carbon centers, which could be valuable for the synthesis of related natural products and their derivatives.

## Supporting Information

File 1 Experimental procedures and characterization data of new compounds.

## Data Availability

All data that supports the findings of this study is available in the published article and/or the supporting information of this article.
