# Supplementary material for: Total synthesis of (±)-simonsol C using dearomatization as key reaction under acidic conditions
Source: Beilstein J Org Chem. 2025 Mar 17;21:601–6. doi: 10.3762/bjoc.21.47 (PMC11931641; doi:10.3762/bjoc.21.47)
Supplement: File 1 — Experimental procedures and characterization data of new compounds. [file Beilstein_J_Org_Chem-21-601-s001.pdf]

BEILSTEIN JOURNAL OF ORGANIC CHEMISTRY

# Supporting Information

for

# Total synthesis of (±)-simonsol C using dearomatization as key reaction under acidic conditions

Xiao-Yang Bi, Xiao-Shuai Yang, Shan-Shan Chen, Jia-Jun Sui, Zhao-Nan Cai, Yong-Ming Chuan and Hong-Bo Qin

*Beilstein J. Org. Chem.* **2025,** *21,* 601–606. doi:10.3762/bjoc.21.47

# Experimental procedures and characterization data of new compounds

License and Terms: This is a supporting information file under the terms of the Creative Commons Attribution License (https://creativecommons.org/licenses/by/4.0). Please note that the reuse, redistribution and reproduction in particular requires that the author(s) and source are credited and that individual graphics may be subject to special legal provisions.
The license is subject to the *Beilstein Journal of Organic Chemistry* terms and conditions: (https://www.beilstein-journals.org/bjoc/terms)

# Table of contents

I. Comparison of the spectra of natural and synthetic simonsol C..................... S2

II. Conditions optimization........................................................................ S4

III. Experimental procedures ...................................................................... S4

IV. NMR spectra for the synthesized compounds........................................... S10

# I. Comparison of the spectra of natural and synthetic simonsol C

**Table S1**. Comparison of the $^{1}$H NMR data recorded for simonsol C obtained by the present route with those reported by Dong [1].

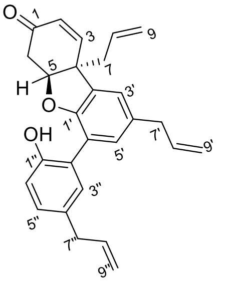


| No. | **Synthetic product ($\delta_H$)** [a] | No. | **Isolated product ($\delta_H$)** [b] | **Δδ** |
|---|---|---|---|---|
| OH-1” | 7.89 (1H, s) | OH-1” | 7.77 (1H, s) | +0.11 |
| 3’ | 7.22 (1H, d, 1.9) | 3’ | 7.23 (1H, d, 1.2) | -0.01 |
| 5’ | 7.12 (1H, d, 1.9) | 5’ | 7.12 (1H, d, 1.2) | 0 |
| 3” | 7.07 (1H, d, 2.3) | 3” | 7.07 (1H, d, 2.1) | 0 |
| 5” | 7.00 (1H, dd, 8.2, 2.1) | 5” | 7.01 (1H, dd, 8.2, 2.1) | -0.01 |
| 6” | 6.86 (1H, d, 8.2) | 6” | 6.86 (1H, d, 8.2) | 0 |
| 3 | 6.71 (1H, dd, 10.2, 1.9) | 3 | 6.71 (1H, dd, 10.2, 1.8) | 0 |
| 8”<br>8’<br>2<br>8 | 6.11-5.81<br>(4H, m) | 8”<br>8’<br>2<br>8 | 6.01(1H, ddt, 18.0, 12.4, 6.6)<br>5.96 (1H, ddt, 13.6, 11.7, 6.8)<br>5.93 (1H, d, 10.2)<br>5.91 (1H, dddd, 16.9, 10.1, 7.8, 6.8) | -- |
| 9 | 5.34 (1H, m)<br>5.17 (1H, dd, 10.1, 2.0) | 9 | 5.29 (1H, dd, 16.9, 2.0)<br>5.18 (1H, dd, 10.1, 2.0) | +0.05<br>-0.01 |
| 9’<br>9”<br>5 | 5.14-4.95<br>(5H, m) | 9’<br>9”<br>5 | 5.11 (1H, dd, 13.6, 3.6)<br>5.08 (1H, dd, 11.7, 3.6)<br>5.03 (1H, ddt, 18.0, 3.6, 1.7)<br>5.02 (1H, ddt, 12.4, 3.6, 1.7)<br>4.99 (1H, ddd, 3.4, 1.8, 1.5) | -- |
| 7’ | 3.39 (2H, d, 6.8) | 7’ | 3.40 (2H, d, 6.8) | -0.01 |
| 7” | 3.30 (2H, dt, 6.6, 1.7) | 7” | 3.30 (2H, dt, 6.6, 1.7) | 0 |
| 7 | 2.97 (1H, dd, 14.0, 6.8) | 7 | 2.97 (1H, dd, 14.0, 6.8) | 0 |
| 7<br>6α | 2.84(2H,dd,8.3, 3.4) | 7<br>6α | 2.79 (1H, dd, 14.0, 7.8)<br>2.89 (1H,dd, 16.0, 3.4) | -- |
| 6β | 2.81（m, 1H） | 6β | 2.83 (1H, dd, 16.0, 1.5) | -0.02 |

[a]Spectrum recorded in $(CD_3)_2CO$ at 400 MHz. [b]Data obtained from literature [1], spectrum recorded in $(CD_3)_2CO$ at 600 MHz.

**Table S2**. Comparison of the $^{13}C$ NMR data recorded for simonsol C obtained by the present route with those reported by Dong [1].

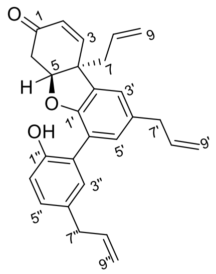


| No. | **Synthetic product** (δc) [a] | No. | **Isolated product** (δc) [b] | Δδ |
|---|---|---|---|---|
| 1 | 195.2 | 1 | 195.1 | +0.1 |
| 1' | 155.4 | 1' | 155.2 | +0.2 |
| 1'' | 153.7 | 1'' | 153.5 | +0.2 |
| 3 | 149.7 | 3 | 149.5 | +0.2 |
| 8'' | 139.2 | 8'' | 139.0 | +0.2 |
| 8' | 139.1 | 8' | 138.9 | +0.2 |
| 4' | 134.4 | 4' | 134.2 | +0.2 |
| 8 | 134.0 | 8 | 133.8 | +0.2 |
| 4'' | 132.9 | 4'' | 132.7 | +0.2 |
| 3'' | 132.1 | 3'' | 131.9 | +0.2 |
| 2' | 132.0 | 2' | 131.8 | +0.2 |
| 5' | 131.8 | 5' | 131.6 | +0.2 |
| 5'' | 129.8 | 5'' | 129.7 | +0.1 |
| 2 | 127.7 | 2 | 127.5 | +0.2 |
| 2'' | 125.1 | 2'' | 124.9 | +0.2 |
| 3' | 123.5 | 3' | 123.3 | +0.2 |
| 6' | 122.4 | 6' | 122.2 | +0.2 |
| 9 | 119.8 | 9 | 119.6 | +0.2 |
| 6'' | 117.4 | 6'' | 117.2 | +0.2 |
| 9' | 115.9 | 9' | 115.7 | +0.2 |
| 9'' | 115.6 | 9'' | 115.5 | +0.1 |
| 5 | 85.9 | 5 | 85.7 | +0.2 |
| 4 | 49.7 | 4 | 49.5 | +0.2 |
| 7 | 40.9 | 7 | 40.7 | +0.2 |
| 7' | 40.5 | 7' | 40.3 | +0.2 |
| 7'' | 40.0 | 7'' | 39.9 | +0.1 |
| 6 | 39.5 | 6 | 39.3 | +0.2 |

[a]Spectrum recorded in $(CD_3)_2CO$ at 100 MHz. [b]Data obtained from literature [1], spectrum recorded in $(CD_3)_2CO$ at 150 MHz.

# II. Conditions optimization

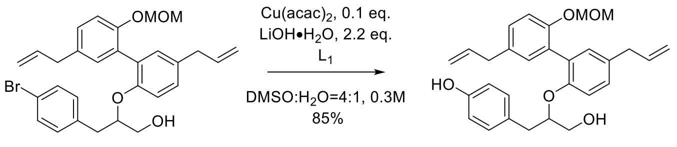


19 20

| entry | equiv | temperature | yield (%) |
|---|---|---|---|
| 1 | 0.05 | 60 °C | 68 |
| 2 | 0.1 | 60 °C | 79 |
| 3 | 0.2 | 60 °C | 77 |
| 4 | 0.1 | 80 °C | 85 |
| 5 | 0.1 | 100 °C | 81 |

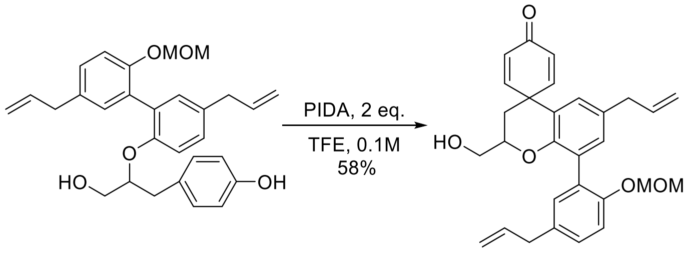


20 14

| entry | time | temperature | yield (%) |
|---|---|---|---|
| 1 | 30 mins | 0 °C | 31 |
| 2 | 30 mins | -78 °C | 42 |
| 3 | 30 mins | -20 °C | 52 |
| 4 | 30 mins | -30 °C | 58 |
| 5 | 15 mins | -30 °C | 42 |
| 6 | 20 mins | -30 °C | 49 |

# III. Experimental procedures

**General experimental procedures:** All reactions were performed with dry solvents under anhydrous conditions unless otherwise noted. Heating reactions involved in the experiments were performed using an oil bath unless specified otherwise. Dry tetrahydrofuran (THF) was distilled over sodium. Dichloromethane (DCM) was distilled over calcium hydride. *N*,*N*-Dimethylformamide (DMF) was dried over calcium hydride. Reagents were used as received without further purification unless otherwise stated. Silica gel (200–300 mesh, Qingdao Marine Chemical Ltd., China), light petroleum ether (bp 60–90 °C), and ethyl acetate were used for product purification by flash column chromatography. Proton nuclear magnetic resonance ($^{1}$H NMR) spectra were recorded on a Bruker Avance 400 spectrometer at 400 MHz. Carbon-13 nuclear magnetic resonance ($^{13}$C NMR) were

recorded on a Bruker Avance 400 at 100 MHz. Data are presented as follows: chemical shift, multiplicity (s = singlet, d = doublet, dd = doublet of doublets, t = triplet, q = quartet, m = multiplet, br = broad, coupling constant (*J*) in hertz (Hz), and integration. Mass spectra were recorded on a VG-AutoSpec-3000 spectrometer. High-resolution mass spectral analysis (HRMS) data were recorded via electrospray ionization (ESI) mass spectrometry using a time-of-flight analyzer.

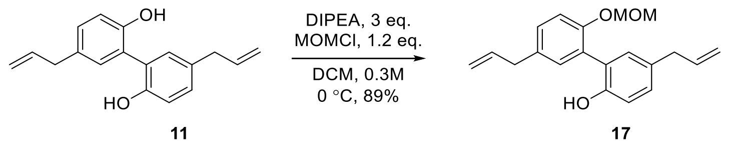


**Synthesis of compound 17:** To a solution of **11** (3.0 g, 11 mmol, 1.0 equiv) in DCM (37.5 mL) was added DIPEA (6.2 mL, 33 mmol, 3 equiv) and the mixture was stirred for 5 minutes at 0 °C. Then, MOMCl (1.0 mL, 14 mmol, 1.2 equiv) was added and stirring continued for 5 h at 0 °C and the reaction was quenched with $NH_4Cl$ aqueous solution. The mixture was concentrated in vacuo, diluted with EtOAc (50 mL), and washed with brine (3 × 10 mL). The organic phase was dried over $Na_2SO_4$ and concentrated in vacuo. The crude product was purified by column chromatography to provide compound **17** (3.1 g, 89%) as colorless liquid. $^1$**H NMR** (400 MHz, $CDCl_3$) δ 7.21 – 7.04 (m, 5H), 6.96 (d, J = 8.2 Hz, 1H), 6.09 (s, 1H), 5.98 (m, 2H), 5.17 – 5.03 (m, 6H), 3.38 (q, J = 5.3, 3.6 Hz, 7H). $^{13}$**C NMR** (100 MHz, CDC13) δ 152.0, 151.9, 137.9, 137.4, 135.4, 132.6, 132.5, 131.3, 129.5, 129.5, 128.4, 126.2, 117.4, 116.7, 116.2, 115.7, 96.2, 77.5, 77.2, 76.8, 56.6, 39.6.

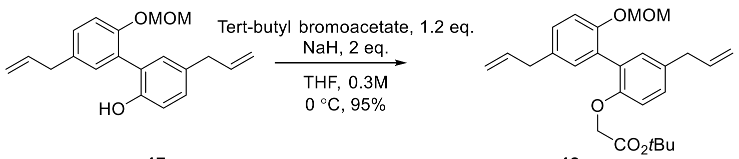


**Synthesis of compound 18:** To a solution of **17** (2.6 g, 8.4 mmol, 1.0 equiv) in 9 mL of THF cooled to 0 °C, a suspension of 60% NaH (0.67 g, 17 mmol, 2 equiv) in 19 mL of THF was added and the mixture stirred for 30 minutes at 0 °C. Then, *tert*-butyl bromoacetate (1.5 mL, 10 mmol, 1.2 equiv) was added and stirring continued for 2 h at 0 °C. The reaction was quenched with water. The mixture was concentrated in vacuo and diluted with EtOAc (50 mL), and washed with brine (3 × 10 mL). The organic phase was dried over $Na_2SO_4$ and concentrated in vacuo. The crude product was purified by column chromatography to provide compound **18** (3.4 g, 95%) as colorless liquid. $^1$**H NMR** (400 MHz, $CDCl_3$)

δ 7.22 – 7.10 (m, 5H), 6.84 – 6.77 (m, 1H), 6.02 (m, 2H), 5.19 – 5.05 (m, 6H), 4.45 (s, 2H), 3.44 – 3.34 (m, 7H), 1.48 (s, 9H). **$^{13}$C NMR** (100 MHz, $CDCl_3$) δ 168.3, 154.1, 153.5, 137.8, 137.7, 133.3, 132.8, 132.0, 131.7, 128.8, 128.7, 128.4, 128.4, 115.8, 115.7, 115.7, 112.5, 95.6, 81.9, 77.5, 77.2, 76.8, 66.7, 55.8, 39.5, 39.4, 28.1. HRMS ($ESI^+$): $C_{26}H_{36}O_5$ $[M+NH_4]^+$ calcd.442.2588, found 442.2579.

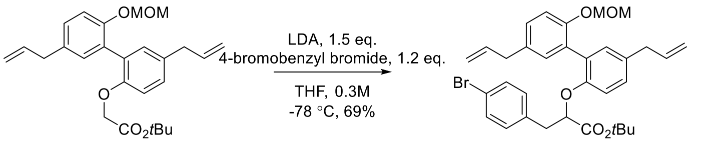


**18** **16**

**Synthesis of compound 16:** To a solution of **18** (3.0 g, 7.1 mmol, 1.0 equiv) in 14 mL of THF cooled to −78 °C, a suspension of 1 M LDA (11 ml, 11 mmol, 1.5 equiv) was added and the mixture stirred for 1 h. Then, a solution of 4-bromobenzyl bromide (2.1 g, 8.5 mmol, 1.2 equiv) in THF was added and stirring continued for 2 h at −78 °C. The reaction was quenched with water. The mixture was concentrated in vacuo, diluted with EtOAc (50 mL), and washed with brine (3 × 10 mL). The organic phase was dried over $Na_2SO_4$ and concentrated in vacuo. The crude product was purified by column chromatography to provide compound **16** (2.9 g, 69%) as colorless liquid. **$^1$H NMR** (400 MHz, $CDCl_3$) δ 7.24 – 7.11 (m, 4H), 7.08 – 6.98 (m, 3H), 6.72 – 6.60 (m, 3H), 6.08 – 5.83 (m, 2H), 5.20 – 4.95 (m, 6H), 4.47 (m, 1H), 3.43 – 3.26 (m, 7H), 2.91 (dd, $J$ = 6.0, 3.6 Hz, 2H), 1.36 (s, 9H). **$^{13}$C NMR** (100 MHz, $CDCl_3$) δ 170.0, 153.6, 153.5, 137.8, 137.7, 135.3, 133.2, 132.5, 131.9, 131.8, 131.5, 131.0, 128.5, 128.4, 128.2, 115.9, 115.7, 115.6, 112.4, 95.6, 82.0, 77.8, 55.9, 39.6, 39.4, 38.4, 28.0. HRMS ($ESI^+$): $C_{33}H_{41}BrNO_5$ $[M+NH_4]^+$ calcd. 610.2163, found 610.2147;

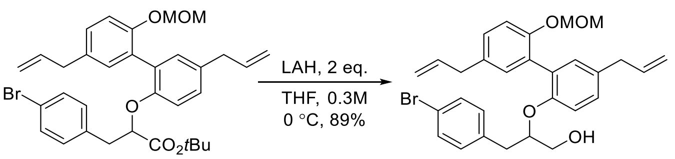


**16** **19**

**Synthesis of compound 19:** To a solution of **16** (1.2 g, 2.0 mmol, 1.0 equiv) in 7 mL of THF cooled to 0 °C, LAH (0.15 g, 4.1 mmol, 2 equiv) was added and stirring continued for 10 minutes at 0 °C. The reaction was quenched with water. The mixture was concentrated in vacuo, diluted with EtOAc (50 mL), and washed with brine (3 × 10 mL). The organic phase was dried over $Na_2SO_4$ and concentrated in vacuo. The crude product was purified by column chromatography to provide compound **19** (0.93g, 89%) as colorless liquid. **$^1$H NMR** (400 MHz, $CDCl_3$) δ 7.35 – 7.25 (m, 2H), 7.16 (dd, $J$ = 8.3, 2.3 Hz,

1H), 7.13 – 7.04 (m, 4H), 6.94 – 6.89 (m, 2H), 6.81 (d, *J* = 9.1 Hz, 1H), 5.97 (m, 2H), 5.14 – 5.02 (m, 4H), 4.99 (d, *J* = 6.5 Hz, 1H), 4.90 (d, *J* = 6.5 Hz, 1H), 4.38 (s, 1H), 3.69 (d, *J* = 12.0 Hz, 1H), 3.45 – 3.30 (m, 5H), 3.20 (s, 3H), 2.91 (dd, *J* = 13.8, 6.2 Hz, 1H), 2.74 (dd, *J* = 13.8, 7.1 Hz, 1H). **$^{13}$C NMR** (100 MHz, $CDCl_3$) δ 153.1, 153.0, 137.6, 137.6, 136.6, 134.7, 132.7, 131.8, 131.5, 131.4, 131.28, 128.9, 128.7, 115.9, 115.7, 96.8, 77.4, 77.1, 76.8, 62.4, 56.2, 39.5, 39.4, 36.3. HRMS (ESI$^+$): $C_{29}H_{35}BrNO_4$ $[M+NH_4]^+$ calcd.540.1744 found 540.1732.

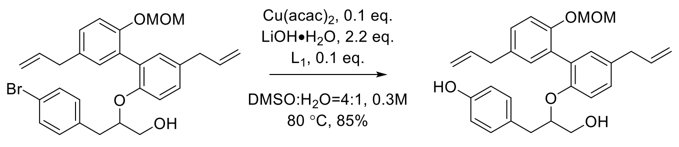


**19** **20**

**Synthesis of compound 20:** Compound **19** (58 mg, 1.1 mmol, 1 equiv), $LiOH{\cdot}H_2O$ (0.10 g, 2.4 mmol, 2.2 equiv), $Cu(acac)_2$ (29 mg, 0.1 mmol, 0.1 equiv) and ligand $L_1$ (36 mg, 0.1 mmol, 0.1 equiv) were placed into a Schlenk tube (10 mL) with a magnetic stirring bar. The reaction vessel was evacuated and backfilled with argon three times, and DMSO/$H_2O$ 4:1 (3.7 mL) was added afterwards. The reaction mixture was heated at 80 °C for 24 h under vigorous stirring. The mixture was concentrated in vacuo, diluted with EtOAc (50 mL), and washed with brine (3 × 10 mL). The organic phase was dried over $Na_2SO_4$ and concentrated in vacuo. The crude product was purified by column chromatography to provide compound **20** (431 mg, 85%) as yellow liquid. **$^1$H NMR** (400 MHz, $CDCl_3$) δ 7.21 – 7.08 (m, 5H), 6.97 – 6.86 (m, 3H), 6.74 – 6.62 (m, 3H), 6.00 (m, 2H), 5.18 – 5.05 (m, 4H), 5.00 (d, *J* = 6.5 Hz, 1H), 4.90 (d, *J* = 6.6 Hz, 1H), 4.43 (s, 1H), 3.71 (d, *J* = 11.6 Hz, 1H), 3.49 (dd, *J* = 11.3, 5.6 Hz, 1H), 3.43 – 3.37 (m, 4H), 3.19 (s, 3H), 2.95 (dd, *J* = 13.9, 5.0 Hz, 1H), 2.69 (dd, *J* = 13.9, 8.6 Hz, 1H). **$^{13}$C NMR** (100 MHz, $CDCl_3$) δ154.8, 153.3, 152.7, 137.7, 137.5, 135.1, 132.6, 131.8, 131.5, 130.8, 130.4, 129.0, 128.9, 128.8, 128.8, 118.2, 115.9, 115.7, 115.4, 97.0, 80.1, 77.4, 77.3, 77.1, 76.8, 62.6, 56.2, 39.5, 39.4, 35.9. HRMS (ESI$^+$): $C_{29}H_{31}O_5$ $[M-H]^-$ calcd. 459.2177, found 459.2170.

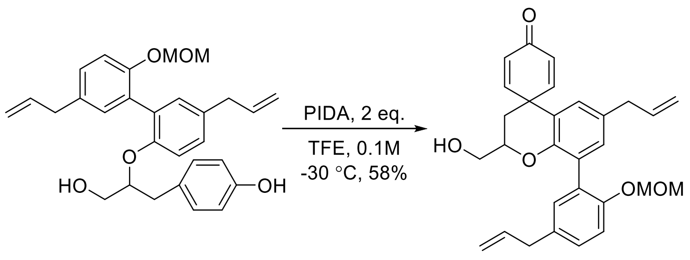


**Synthesis of compound 14:** A solution of compound **20** (430 mg, 0.935 mmol, 1.0 equiv) in 14 mL of TFE was cooled to −30 °C and stirred for 5 minutes. Then, a solution of PIDA (278 mg, 1.12 mmol, 1.2 equiv) in TFE was added to the flask and stirring continued for 0.5 h at −30 °C. The reaction was quenched with water. The mixture was concentrated in vacuo, diluted with EtOAc (50 mL), and washed with brine (3 × 10 mL). The organic phase was dried over $Na_2SO_4$ and concentrated in vacuo. The crude product was purified by column chromatography to provide compound **14** (248 mg, 58%) as colorless liquid [2].$^1$**H NMR** (400 MHz, $CDCl_3$) δ 7.23 – 7.00 (m, 5H), 6.88 (dd, *J* = 10.0, 3.0 Hz, 1H), 6.73 (d, *J* = 2.2 Hz, 1H), 6.46 (dd, *J* = 10.0, 1.9 Hz, 1H), 6.26 (dd, *J* = 10.1, 1.9 Hz, 1H), 6.06 – 5.81 (m, 2H), 5.19 – 4.88 (m, 6H), 4.46 – 4.27 (m, 1H), 3.84 (dd, *J* = 12.1, 2.9 Hz, 1H), 3.58 (dd, *J* = 12.0, 4.4 Hz, 1H), 3.39 (d, *J* = 6.9 Hz, 2H), 3.27 (d, *J* = 6.9 Hz, 5H), 2.37 (t, *J* = 12.9 Hz, 1H), 1.83 (dd, *J* = 13.7, 2.4 Hz, 1H).$^{13}$**C NMR** (100 MHz, $CDCl_3$) δ 185.7, 154.1, 152.9, 152.3, 149.6, 137.5, 137.2 134.6, 132.3, 131.5, 131.4, 129.4, 129.1, 129.0, 128.4, 127.6, 126.6, 119.5, 116.0, 115.9, 96.6, 64.6, 56.1 41.8, 39.5, 39.2, 34.0.

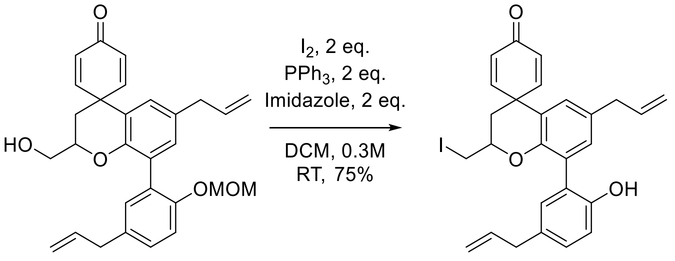


**Synthesis of compound 15:** After stirring a solution of imidazole (30 mg, 0.44 mmol, 2.0 equiv) and $PPh_3$ (115 mg, 0.44 mmol, 2.0 equiv) in DCM (1 mL) at room temperature for 0.5 h, iodine (112 mg, 0.44 mmol, 2 equiv) was added and the resulted solution was stirred at room temperature for 0.5 h. Then, compound **14** (100 mg, 0.22 mmol, 1.0 equiv) in 1 mL DCM was added dropwise and stirring was continued overnight until the starting material was consumed (judged by TLC). The reaction was quenched with saturated $Na_2S_2O_3$ solution (3 mL) and extracted with ethyl acetate (3 × 5 mL). The combined organic phases were washed with brine (3 × 10 mL), dried over $Na_2SO_4$, and concentrated

under vacuum. The crude product was purified by column chromatography to obtain compound **15** (86 mg, 75%) as white solid.

**$^1$H NMR** (400 MHz, $CDCl_3$) δ 7.17 – 7.09 (m, 2H), 7.07 (dd, *J* = 7.8, 2.2 Hz, 2H), 6.98 (d, *J* = 8.3 Hz, 1H), 6.86 (dd, *J* = 10.0, 3.1 Hz, 1H), 6.77 (d, *J* = 2.2 Hz, 1H), 6.48 (dd, *J* = 9.9, 1.9 Hz, 1H), 6.28 (dd, *J* = 10.0, 1.9 Hz, 1H), 6.00 (ddt, *J* = 16.8, 10.0, 6.7 Hz, 1H), 5.86 (ddt, *J* = 17.4, 9.6, 6.7 Hz, 1H), 5.59 (s, 1H), 5.14 – 4.97 (m, 4H), 4.21 – 4.14 (m, 1H), 3.46 – 3.34 (m, 4H), 3.28 (d, *J* = 6.7 Hz, 2H), 2.26 (dd, *J* = 13.8, 11.4 Hz, 1H), 2.13 (dd, *J* = 13.8, 2.0 Hz, 1H). **$^{13}$C NMR** (100 MHz, $CDCl_3$) δ 185.7, 154.1, 153.2, 152.5, 137.8, 137.1, 133.1, 132.4, 132.0, 129.0, 128.8, 127.6, 126.5, 119.0, 116.0, 115.6, 115.3, 95.4, 71.1, 56.0, 42.1, 39.47, 39.2, 38.1, 29.7, 7.4.

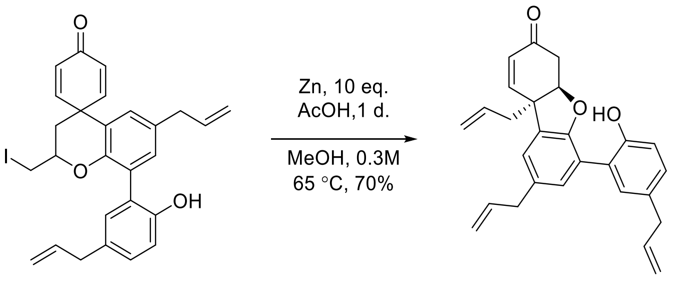


**15** **(±)-Simonsol C**

**Synthesis of compound (±)-simonsol C:** Zinc powder (53 mg, 0.82 mmol, 10.0 equiv) and acetic acid (1 drop) were added to a solution of compound **15** (43 mg, 0.08 mmol, 1.0 equiv) in methanol (1 mL). After stirring at room temperature for 0.5 h, the reaction mixture was refluxed for 1 h until the starting material was consumed. The reaction mixture was subsequently concentrated under reduced pressure and purified by column chromatography, yielding (±)-simonsol C (22 mg, 70% yield) as white amorphous substance. **$^1$H NMR** (400 MHz, acetone-$d_6$) δ 7.89 (s, 1H), 7.22 (d, *J* = 1.9 Hz, 1H), 7.12 (d, *J* = 1.9 Hz, 1H), 7.07 (d, *J* = 2.3 Hz, 1H), 7.00 (dd, *J* = 8.3, 2.2 Hz, 1H), 6.86 (d, *J* = 8.2 Hz, 1H), 6.71 (dd, *J* = 10.2, 1.9 Hz, 1H), 6.11 – 5.81 (m, 4H), 5.34 – 5.24 (m, 1H), 5.17 (dd, *J* = 10.2, 2.1 Hz, 1H), 5.14 – 4.95 (m, 5H), 3.39 (d, *J* = 6.8 Hz, 2H), 3.29 (d, *J* = 6.8 Hz, 2H), 2.90 (d, *J* = 3.9 Hz, 1H), 2.84 (dd, *J* = 8.3, 3.4 Hz, 2H), 2.81 – 2.74 (m, 1H). **$^{13}$C NMR** (100 MHz, acetone-$d_6$) δ 195.2, 149.7, 139.2, 139.1, 134.0, 132.1, 131.8, 129.8, 127.7, 123.5, 119.8, 117.4, 115.9, 115.6, 85.9, 49.7, 40.9, 40.5, 40.1, 39.5.

# IV. NMR spectra for the synthesized compounds

**$^{1}H$ NMR (400 MHz, $CDCl_3$) of compound 17**

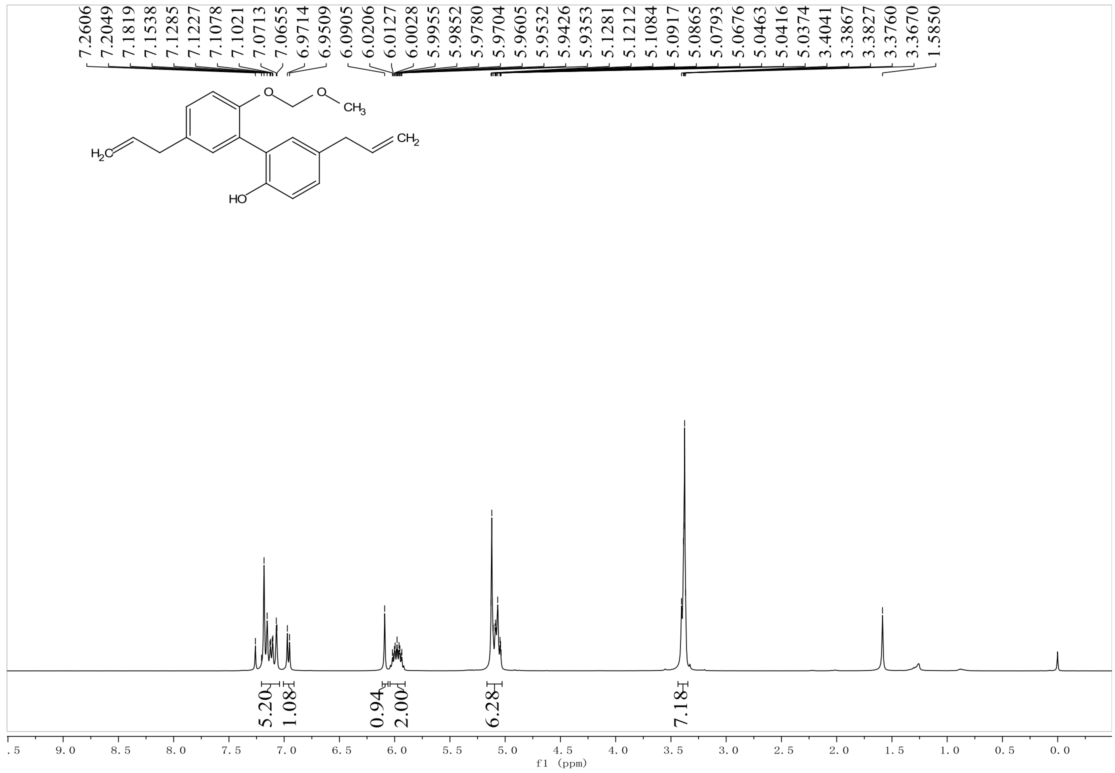


**$^{13}C$ NMR (100 MHz, $CDCl_3$) of compound 17**

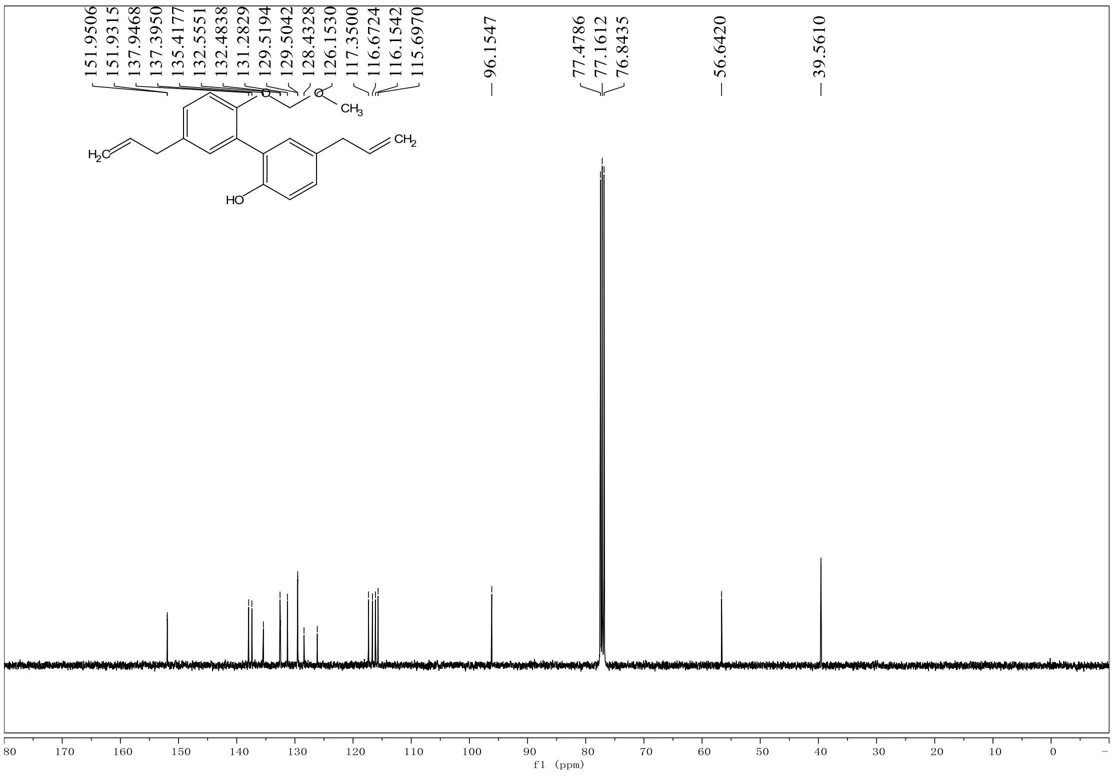

## $^{1}$H NMR (400 MHz, $CDCl_3$) of compound 18

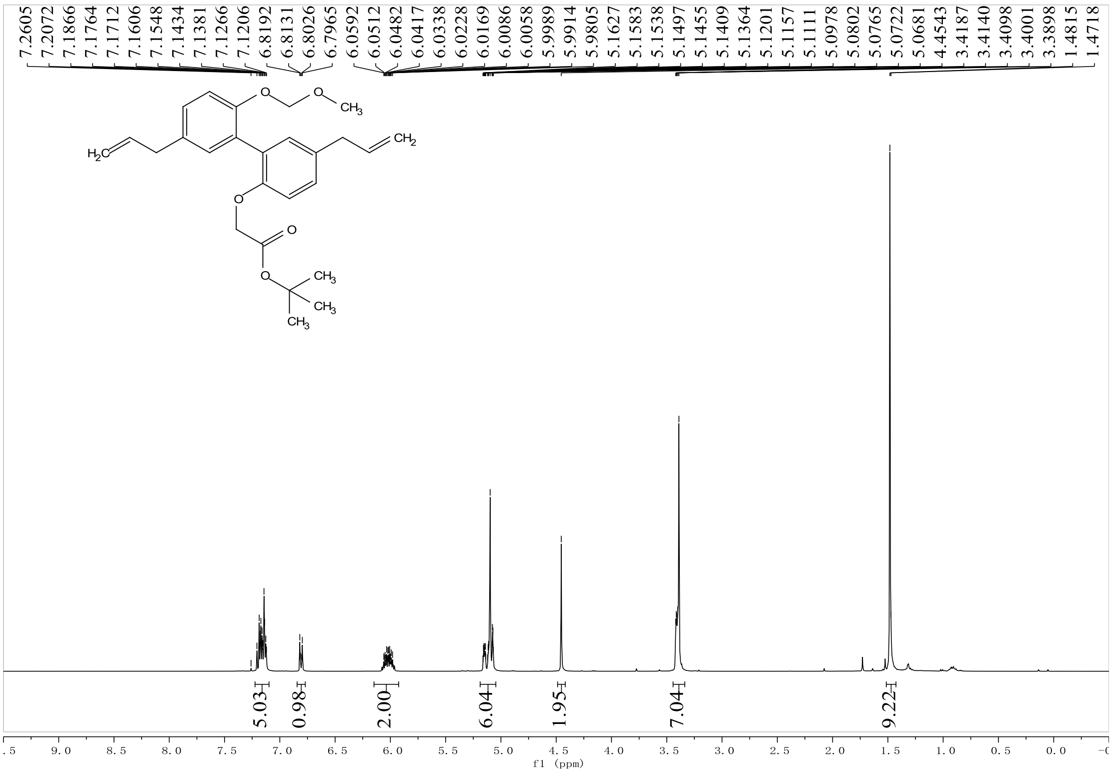


## $^{13}$C NMR (100 MHz, $CDCl_3$) of compound 18

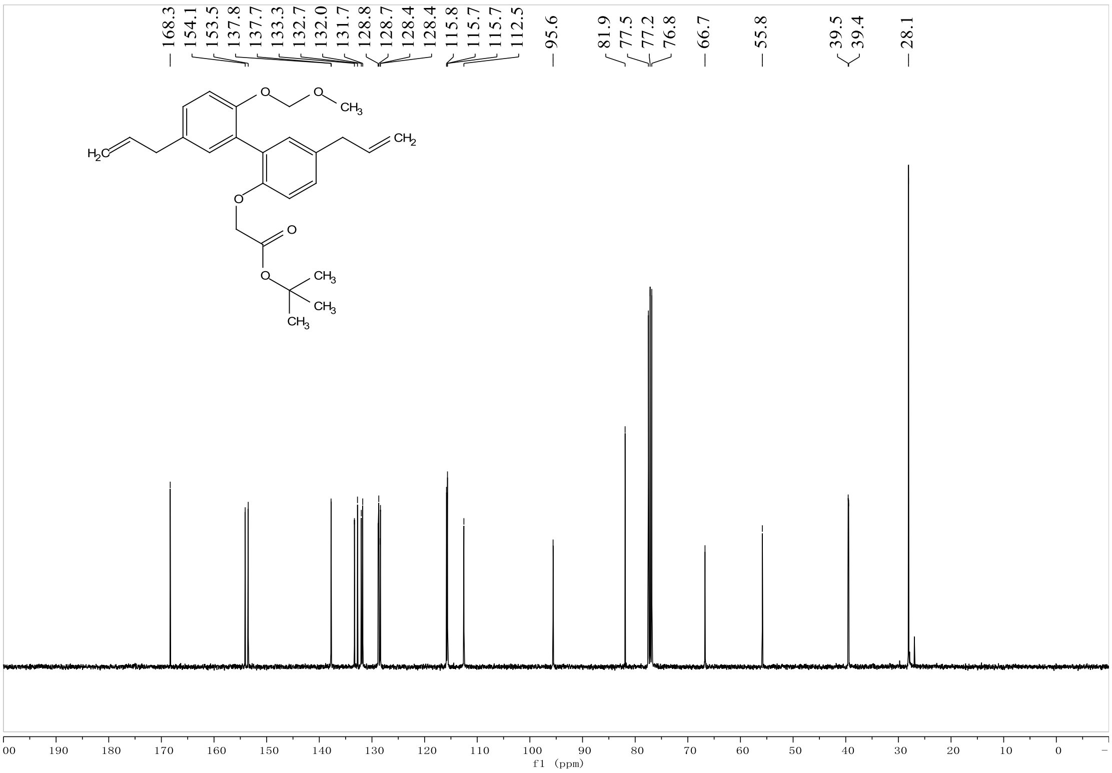

## $^1H$ NMR (400 MHz, CDCl3) of compound 16

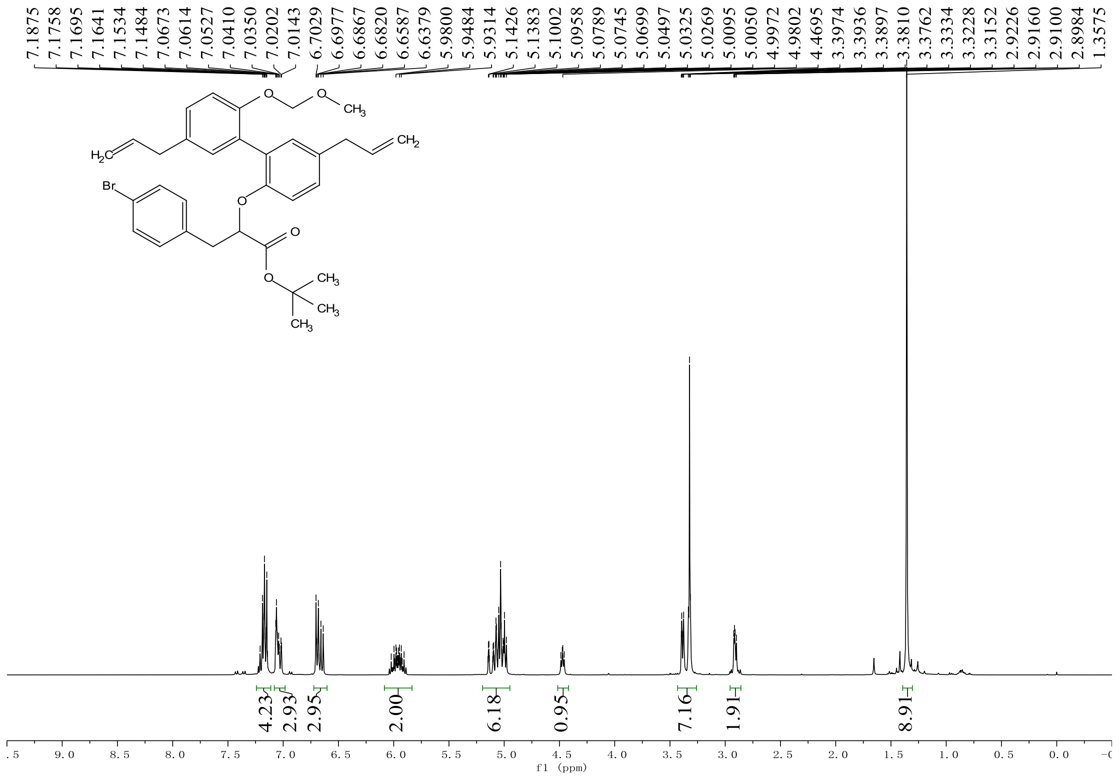

## $^{13}C$ NMR (100 MHz, CDCl3) of compound 16

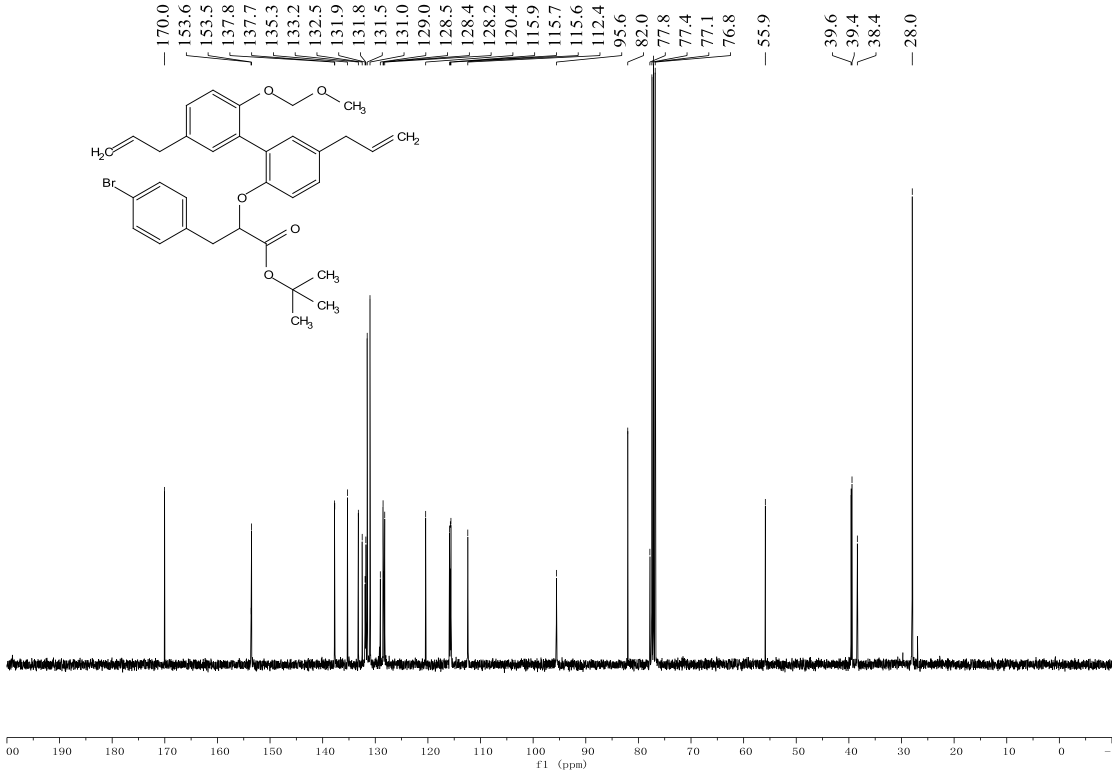

## $^{1}$H NMR (400 MHz, CDCl3) of compound 19

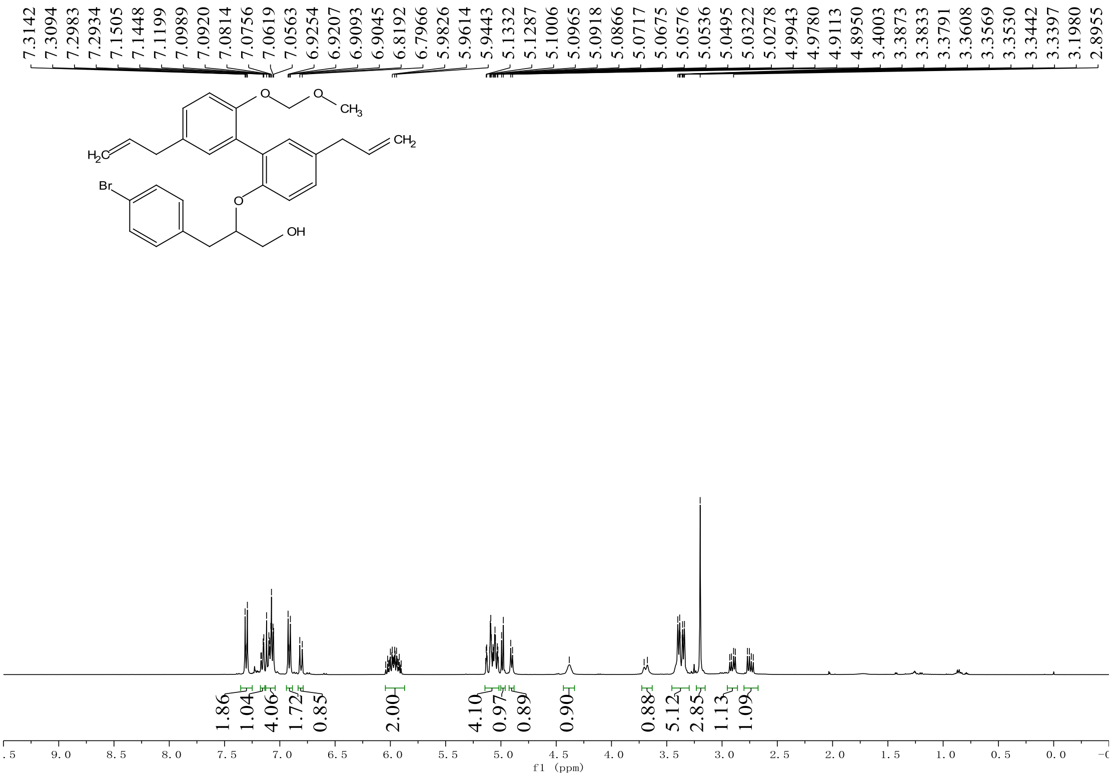


## $^{13}$C NMR (100 MHz, CDCl3) of compound 19

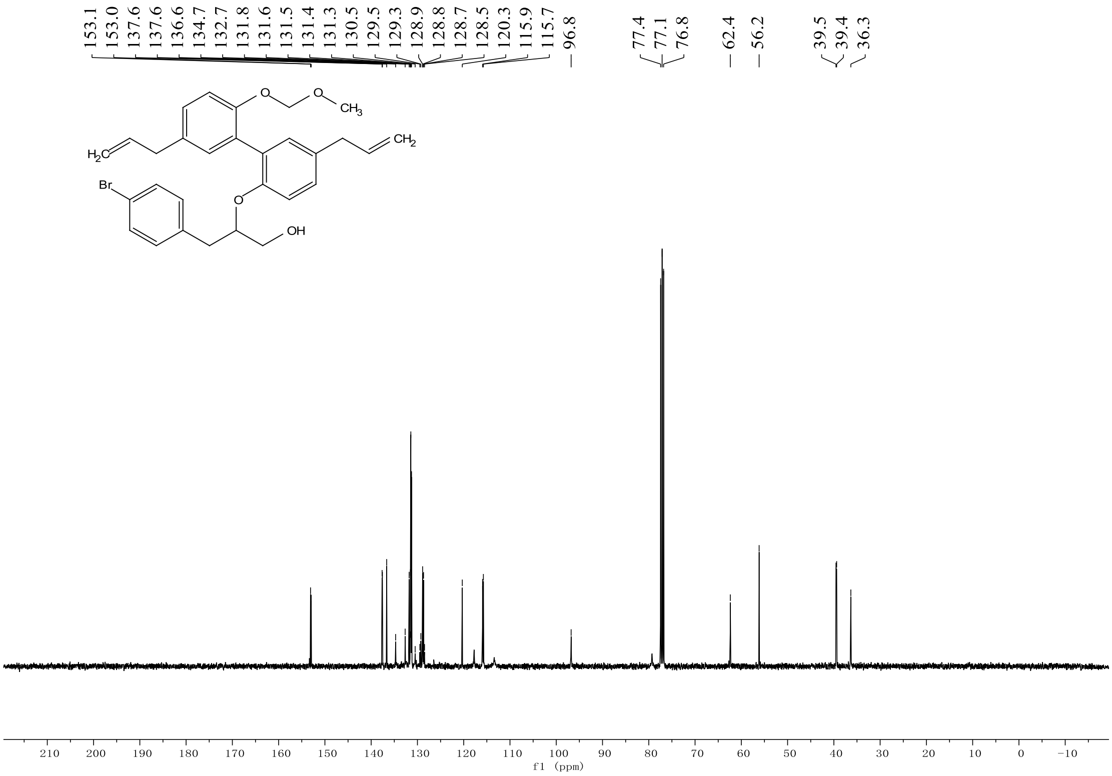

## $^{1}H$ NMR (400 MHz, $CDCl_3$) of compound 20

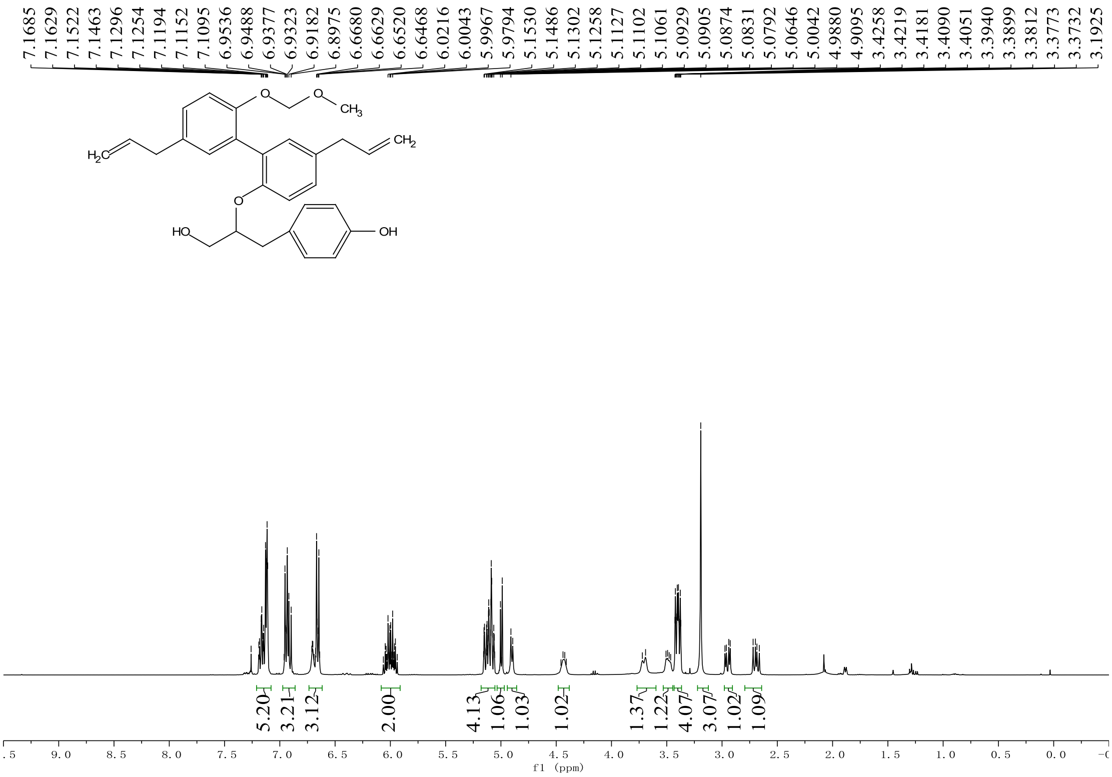


## $^{13}C$ NMR (100 MHz, $CDCl_3$) of compound 20

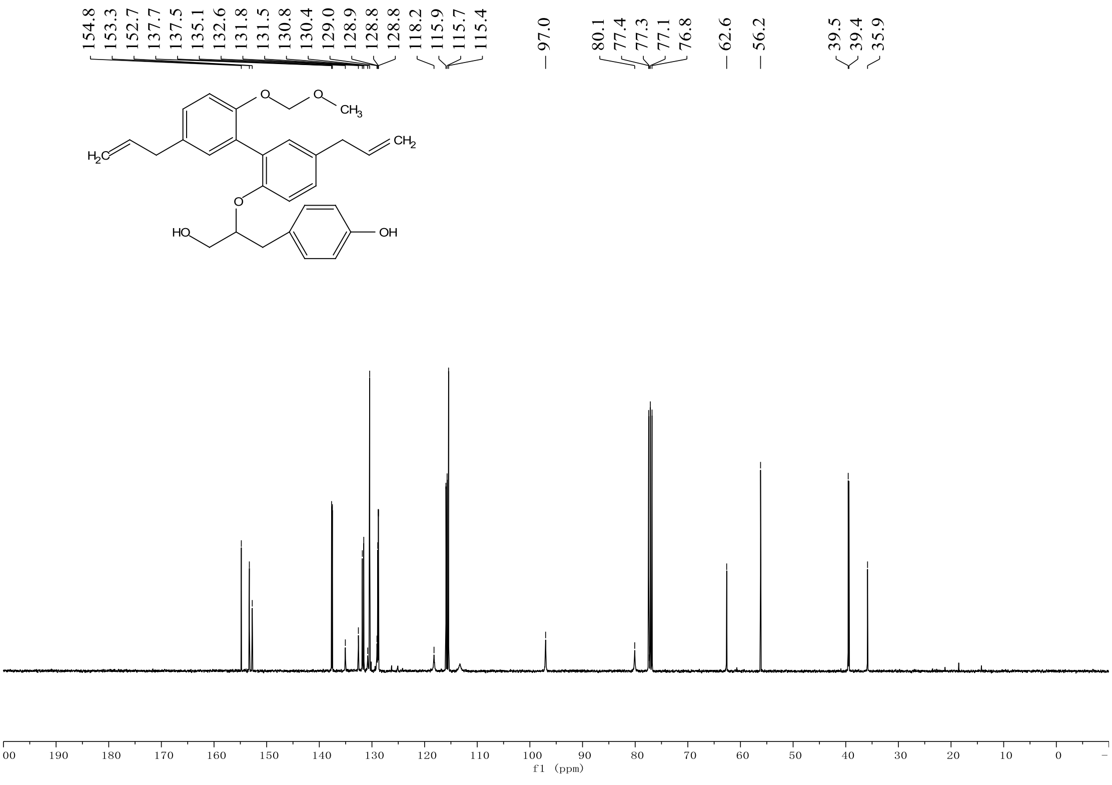

## $^{1}H$ NMR (400 MHz, $CDCl_3$) of compound 14

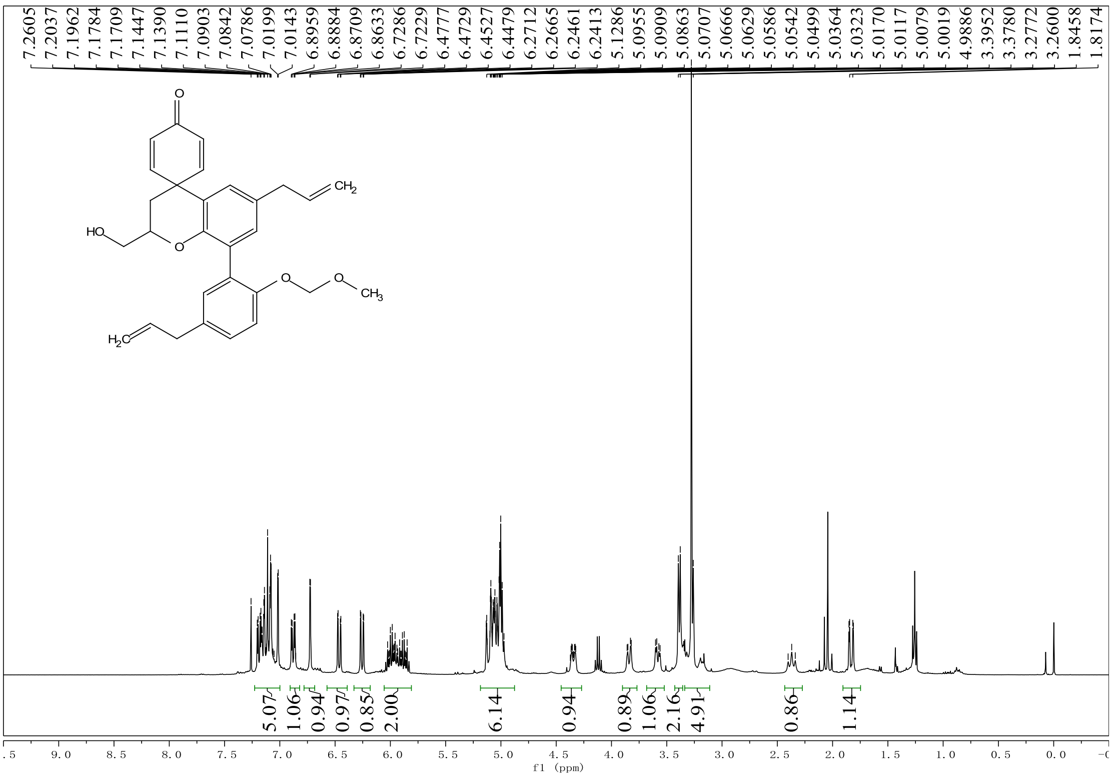


## $^{13}C$ NMR (100 MHz, $CDCl_3$) of compound 14

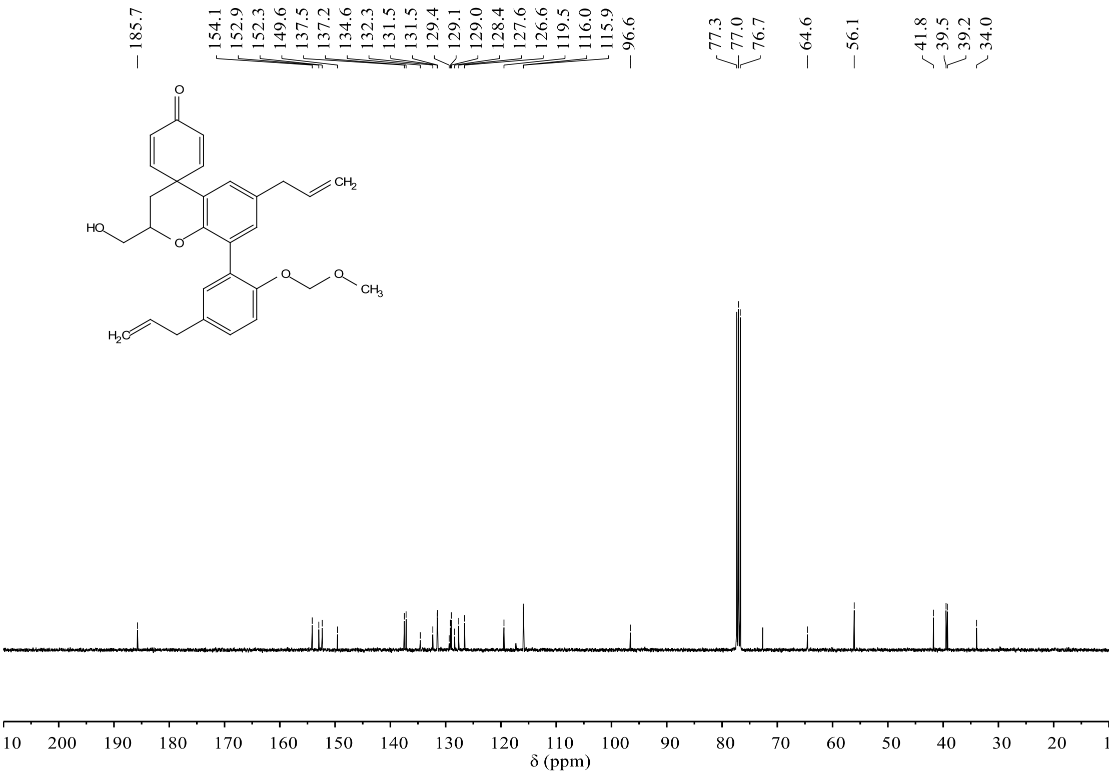

## $^{1}$H NMR (400 MHz, $CDCl_3$) of compound 15

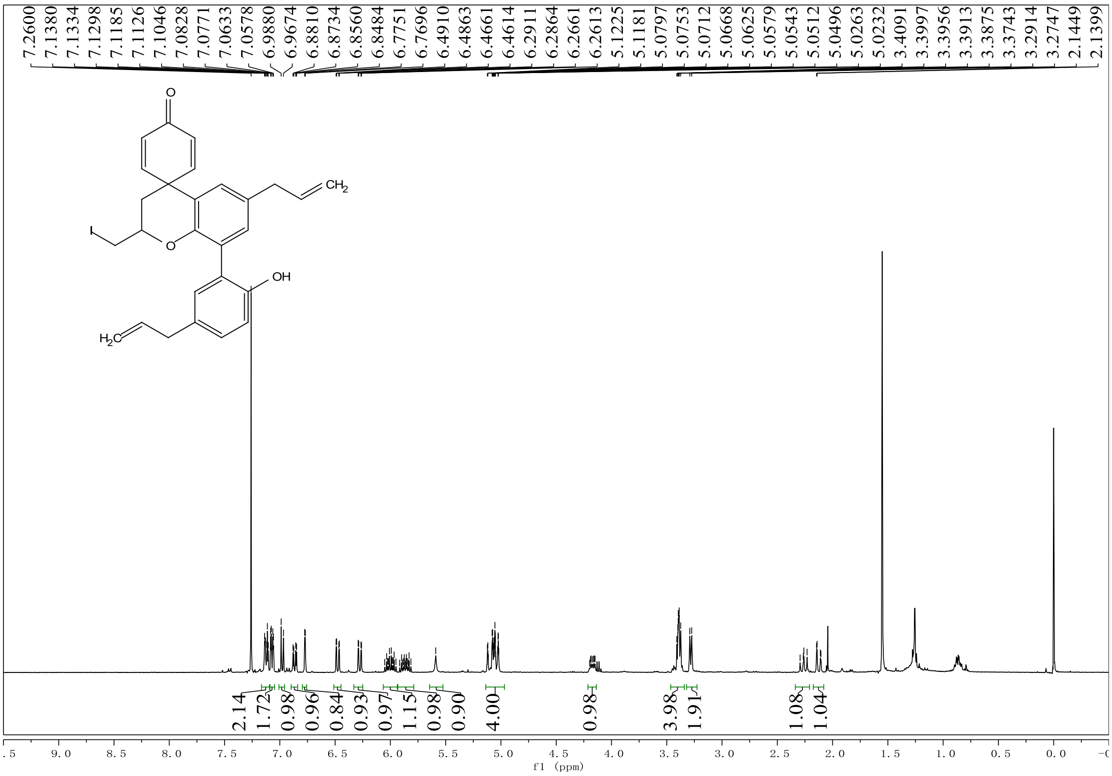


## $^{13}$C NMR (100 MHz, $CDCl_3$) of compound 15

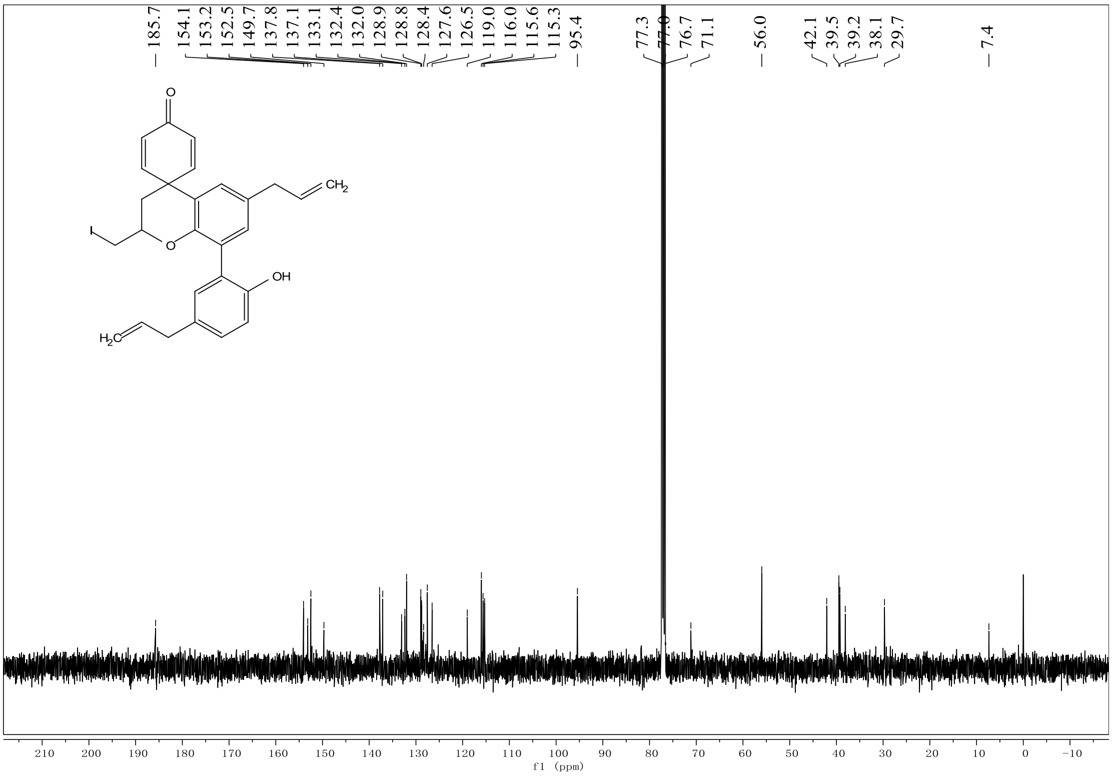

## $^1H$ NMR (400 MHz, $(CD_3)_2CO$) of (±)-simonsol C

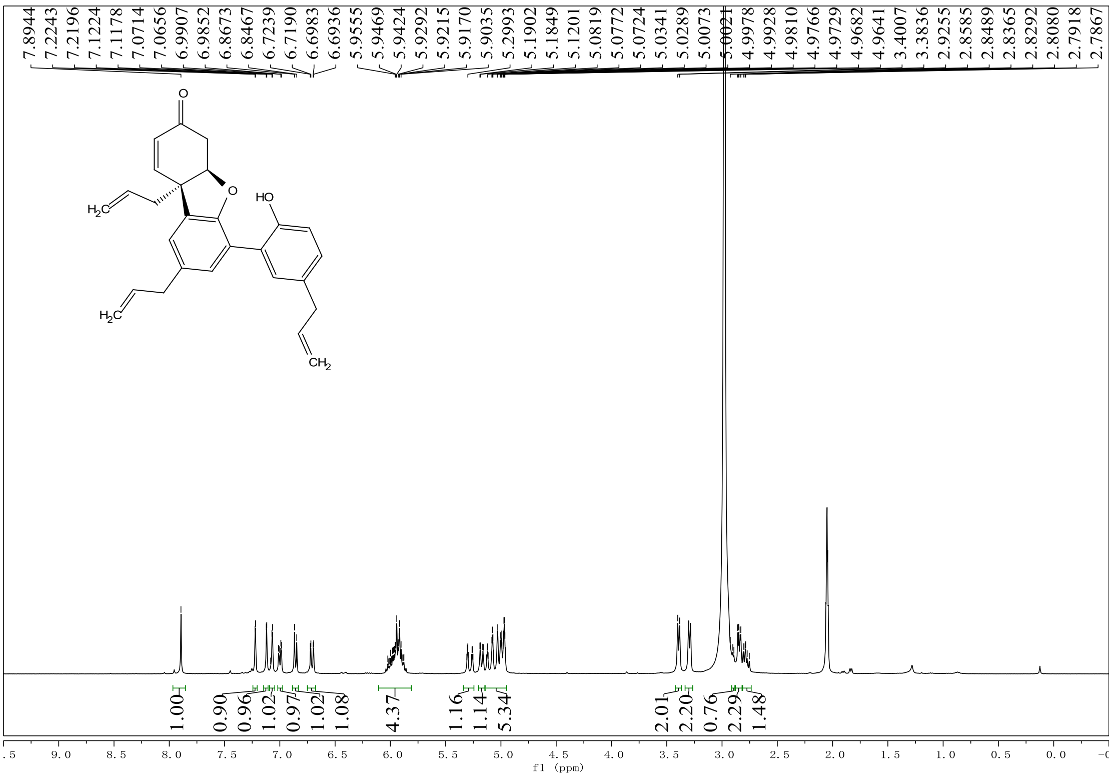


## $^{13}C$ NMR (100 MHz, $(CD_3)_2CO$) of (±)-simonsol C

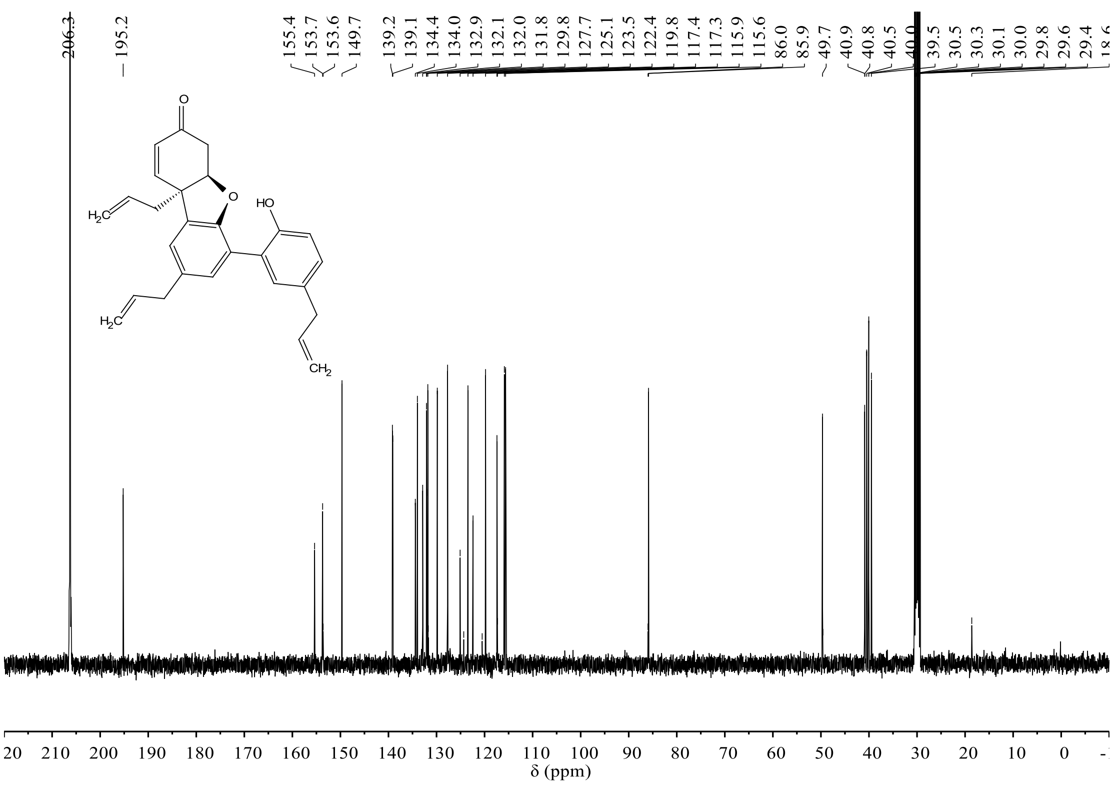



[1] C. Dong, L. Liu, X. Li, Z. Guan, H. Luo and Y. Wang, *Planta Med.* **2013**, *79*, 338-347.

[2] Sui, J.-J.;Wang, K.;Luo, S.-J.;Guo, K.;Yuan, M.-W., Qin, H.-B., *J. Org. Chem.* **2024**, *89*, 7821-7827.
